# Updating and validating the Domestic and Community Skills Assessment

**DOI:** 10.1111/1440-1630.70100

**Published:** 2026-06-16

**Authors:** Neil Woodroffe, Kate E. Webster, Siann Bowman

**Affiliations:** ^1^ School of Allied Health, Human Services and Sport La Trobe University Bundoora Victoria Australia

**Keywords:** DACSA, DACSA‐3, Domestic and Community Skills Assessment, IADL, instrumental activities of daily living, occupational therapy assessment

## Abstract

**Introduction:**

This study outlines the revision and content validity process for the Domestic and Community Skills Assessment, Third Edition (DACSA‐3). The DACSA‐3 is an occupational therapy instrumental activities of daily living (IADL) assessment, which can be used to assess a person with a mental health condition.

**Methods:**

The DACSA‐3 was developed by considering past development and research, technology changes, and current occupational therapy theories. Nine content experts (occupational therapists with mental health experience) were involved in a qualitative and quantitative review of the DACSA‐3 to establish content validity using the content validity ratio (CVR) and content validity index (CVI), with recommended minimum thresholds of 0.78.

**Consumer and community involvement:**

The DACSA‐3 was reviewed by two content reviewers (professionals with lived mental health experience) to ensure that recovery oriented language was included.

**Results:**

The Initial Interview was retained and updated. The Supporting Interview was removed, because it lacked construct validity. The Observation Checklist was renamed to Context List and updated to include environmental and personal factors that may influence task performance. The Objective Assessment subtests were updated and reduced from 17 to 14 subtests. The money handling, personal presentation, and postage handling subtests were removed, because they were no longer relevant or did not fit the occupational category of IADL. The scoring criteria for each subtest were improved by aligning with the rating scale definitions to prevent discrepancies between ratings and clinical judgement. The CVR of DACSA‐3 items ranged from 0.78 to 1.00, and the CVI of the DACSA‐3 was 0.98.

**Conclusion:**

The DACSA‐3 is a revised, contemporary occupational therapy assessment of IADL, which has content validity. The CVR and CVI exceeded the minimum recommended thresholds, and thus, the content validity of the DACSA‐3 was established. Modifications were made to the DACSA‐3 to reduce the administration time and it no longer contains a screening tool.

## PLAIN LANGUAGE SUMMARY

This study describes how the Domestic and Community Skills Assessment, Third Edition (DACSA‐3) was updated. The DACSA‐3 is an occupational therapy test. It can be used to measure the living skills of people with a mental health condition. The update considered earlier research, changes in tasks over time, and current occupational therapy theories. Two content reviewers checked the test to ensure current mental health language was used. Nine occupational therapists then reviewed it step by step to confirm that it was a good measurement of living skills. The Initial Interview was kept and updated. The Supporting Interview was removed because it was not considered a good measurement. The Observation Checklist was renamed the Context List and updated. The subtests were reduced from 17 to 14. The money handling, personal presentation, and postage handling subtests were removed. They were no longer relevant or did not fit with other living skills. The scoring was also improved to reduce differences between ratings and therapist judgement. Overall, the DACSA‐3 is a current test of living skills. It takes less time to complete and no longer includes a screening tool.

**Key Points for Occupational Therapy**

- The Domestic and Community Skills Assessment is an occupational therapy instrumental activities of daily living assessment for mental health.
- Instrumental activities of daily living assessments should be developed considering research, technology, and practice theories.
- Out‐of‐date instrumental activities of daily living assessments should be revised and validated.

## 1 INTRODUCTION

This study outlines the process of updating and validating the Domestic and Community Skills Assessment (DACSA). The DACSA is an occupational therapy instrumental activities of daily living (IADL) assessment, which can be used to assess the domestic and community skills of a person with a mental health condition (Collister & Alexander, 1991; Woodroffe et al., 2025). The Domestic and Community Skills Assessment, Initial Edition (DACSA‐1), was first developed by Collister, Alexander, and Wood in 1987 in response to de‐institutionalisation of Victorian psychiatric facilities (Collister, 1994; Collister & Alexander, 1991; Jaccoud‐Alexander, 1993). It was the role of occupational therapists to assess if patients could safely perform IADL, so they could be moved from psychiatric facilities into the community (Collister, 1994; Collister & Alexander, 1991; Jaccoud‐Alexander, 1993). The term IADL was first named by Lawton and Brody (1969), which involved a more complex set of activities (e.g. phone use, shopping, food preparation, housekeeping, laundry, transportation, medication, and finances) than activities of daily living (ADL) (e.g. toilet, feeding, dressing, grooming, ambulation, and bathing).

There were concerns that people with a mental health condition who had experienced long‐term hospitalisation may experience difficulties surviving in the community due to difficulties performing IADL (Collister, 1994; Jaccoud‐Alexander, 1993). People with a severe mental health condition such as schizophrenia, major depression, and bipolar affective disorder often experience difficulties completing IADL due to associated cognitive deficits (Cambridge et al., 2018; Decker et al., 2017; Gildengers et al., 2007; Kiosses & Alexopoulos, 2005; Lipskaya et al., 2011; Viertiö et al., 2010).

The DACSA‐1 was being used in approximately 20 occupational therapy departments in Victorian psychiatric facilities by 1989 (Collister, 1994; Collister & Alexander, 1991; Jaccoud‐Alexander, 1993). The DACSA‐1 was revised in 1991 to the Domestic and Community Skills Assessment, Research Edition (DACSA‐R), and research was commenced into the reliability and validity of the DACSA‐R (Collister, 1994; Collister & Alexander, 1991; Jaccoud‐Alexander, 1993). The research conducted by Collister (1994) and Jaccoud‐Alexander (1993) was not published in a database or journal and remains archived in the La Trobe University Library in Australia.

The DACSA‐R was made up of three components including an initial interview, supporting interview, and 17 subtests (Collister & Alexander, 1991). The domestic and community skills assessed included “information form completion, money handling, banking, budgeting, bill paying, meal planning, grocery shopping, meal preparation, house cleaning, laundry, personal presentation, medication management, simple first aid, making and keeping appointments, public transport, telephone use, and postal services” (Collister & Alexander, 1991, pp. 5–6). The DACSA‐R contained a “four point, ordinal, unidimensional rating scale” to rate task performance from fully independent to fully dependent (Collister & Alexander, 1991, p. 6).

The DACSA‐R was being used by occupational therapists throughout Australia in the field of mental health including Victoria, Queensland, and New South Wales (Hitch, 2009; Munro et al., 2007; Queensland Health, 2017; Scanlan & Still, 2013). It was being used by occupational therapists working in adolescent mental health services, adult mental health services, extended treatment rehabilitation units, community care units, and forensic mental health services (Hardaker, 2011; Hatton, 2004; Munro et al., 2007; Queensland Health, 2017). The DACSA‐R was also being used as an outcome measure by occupational therapists working in mental health including the Better Access to Mental Health, Medicare initiative in Australia (Hitch, 2009; Unsworth, 2000).

Due to changes in technology, some of the DACSA‐R subtests no longer reflect the way that IADL are performed in society (e.g. banking and telephone use) (Grant, 2006; Hitch et al., 2007). A qualitative and quantitative study conducted by Grant (2006) involving 57 occupational therapists working in Victorian Mental Health Services identified that the DACSA‐R needed to be revised and content validity needed to be re‐established for current mental health practice. There is evidence that the DACSA‐R was still being used by occupational therapists in mental health services in Queensland as a functional assessment for Domestic ADL and IADL in 2017, despite it being out‐of‐date (Queensland Health, 2017).

The development of the Domestic and Community Skills Assessment, Third Edition (DACSA‐3) was commenced in response to the DACSA‐R being out‐of‐date and that IADL are considered important for a person with a mental health condition to thrive and survive in the community (Woodroffe et al., 2025). This study will outline the processes involved to develop and establish content validity of the DACSA‐3.

## 2 METHODS

### 2.1 Ethics

Prior to commencing this project, the development of the DACSA‐3 was approved by the original authors of the DACSA‐R in 2021. Ethical approval (HEC22056) to commence content validity of the DACSA‐3 was received from the La Trobe University Human Research Ethics Committee on 3/07/2023.

### 2.2 Positionality statement

At the commencement of this study, the first author was a Master of Applied Science candidate at La Trobe University and had 15 years of experience working as an occupational therapist in mental health settings in Australia. The first author had used the DACSA‐R for over 10 years in clinical practice and held the view that it was a valuable IADL assessment that warranted revision to reflect contemporary practice. The first author was also the revision author of the DACSA‐3. This prior experience and positive appraisal of the DACSA‐R positioned the first author as having an informed but potentially invested perspective in the revision process. As such, it is acknowledged that complete objectivity was not possible. Instead, reflexive practices were employed throughout the study to recognise and critically consider how prior clinical experience, assumptions about the value of the DACSA‐R, and the dual role as both researcher and revision author may have influenced decision making.

The first author had no direct affiliation with the original authors of the DACSA‐R, although the original authors had previous affiliation with La Trobe University, and they chose not to be involved in this study. The second author (principal supervisor, professor) had no prior experience using the DACSA‐R and no previous affiliation with the original authors and therefore contributed an external perspective to the research process. The third author (co‐supervisor, senior lecturer in occupational therapy) had previous experience using the DACSA‐R in mental health practice and had prior professional affiliation with one of the original authors. This positioned the research team as comprising both familiar and less familiar perspectives in relation to the DACSA‐R. All authors contributed to the development and review of the DACSA‐3 and engaged in ongoing discussion regarding decisions made throughout the revision and validation process. While the research team agreed that maintaining elements of the DACSA‐R structure where appropriate would support continuity of the assessment, this position was considered alongside feedback from content experts, professionals with lived experience, and relevant literature to minimise the influence of individual bias.

### 2.3 Planning for development and content validation of the DACSA‐3

Three studies were used to guide the development of the DACSA‐3 (Benson & Clark, 1982; Cronje et al., 2022; McKenzie et al., 1999). Benson and Clark (1982) developed a flowchart for occupational therapy assessment development, which was used in the development of the DACSA‐R (Collister, 1994). Cronje et al. (2022) conducted a systematic review to develop guidelines for the revision of psychological tests. McKenzie et al. (1999) outlined methods for establishing content validity of an assessment. The following steps were planned and carried out:

1. Obtained copies of previous versions of the DACSA.
2. Conducted a literature search regarding previous development and use of the DACSA.
3. Reviewed previous research about the DACSA.
4. Reviewed feedback from occupational therapists who have used the DACSA‐R.
5. Updated the DACSA by considering past development and research, feedback from occupational therapists, technology changes, and current occupational therapy theories regarding IADL.
6. Received feedback from professionals with lived mental health experience to ensure that recovery oriented language is included in the DACSA‐3.
7. Involved content experts (occupational therapists with mental health experience) to review the content of the DACSA‐3, to decide on test items to help ensure that it measures the occupational performance of IADL, and to establish content validity.

### 2.4 Review of DACSA versions

One official version of the DACSA was reviewed:

- A citation for the DACSA‐1 was found, but the resource was unable to be located for a review (Collister et al., 1987, as cited in Collister, 1994; Collister et al., 1987, as cited in Jaccoud‐Alexander, 1993).
- The DACSA‐R was located for a review (Collister, 1994; Collister & Alexander, 1991; Jaccoud‐Alexander, 1993). The DACSA‐R was chosen to be updated to the DACSA‐3 (Collister & Alexander, 1991).

Three unofficial versions of the DACSA were reviewed:

- The DACSA‐R2 First Trial was located for a review (Mitchell, 2010b).
- The DACSA‐R2 Second Trial was located for a review (DACSA‐R2 Second Trial, 2010)
- A DACSA update was located for a review (Central Coast Mental Health OT Dept, 2009).

An unpublished honour's thesis was found about the development of the DACSA‐R2 First Trial (Mitchell, 2010a). Mitchell (2010a) also conducted a small qualitative study involving three occupational therapists and four participants with mental health conditions to trial the DACSA‐R2, and a recommendation was made for a second trial version of the DACSA‐R2 including establishment of validity and reliability. No evidence was found about the validity and reliability of these three unofficial versions of the DACSA and the changes were not justified (Central Coast Mental Health OT Dept, 2009; DACSA‐R2 Second Trial, 2010; Mitchell, 2010b). Each version had technology updates, but these were also out‐of‐date by 2021 (Central Coast Mental Health OT Dept, 2009; DACSA‐R2 Second Trial, 2010; Mitchell, 2010b). These unofficial versions of the DACSA were however initially used by the revision author to generate ideas for the technology updates in the DACSA‐3.

### 2.5 Review of DACSA research

Three unpublished theses of mixed method design were reviewed to guide development of the DACSA‐3:

- Doctoral research conducted by Jaccoud‐Alexander (1993) included assessment revision, content validity, internal consistency, inter‐rater reliability, test–retest reliability, factor analysis, construct validity, concurrent validity, and discriminant validity of the DACSA‐R.
- Masters research conducted by Collister (1994) included assessment revision, content validity, discriminant validity, and predictive validity of the DACSA‐R.
- Honours research conducted by Grant (2006) was a qualitative and quantitative study conducted to review the content validity and clinical utility of the DACSA‐R by 57 occupational therapists working in mental health in Victoria, Australia. This study provided feedback from occupational therapists who have used the DACSA‐R.

### 2.6 Development of the DACSA‐3

Past development and research of the DACSA‐R, technology changes over the past 30 years and current occupational therapy theories regarding IADL were considered while developing the DACSA‐3. There was an aim to include mental health recovery oriented language where possible in the DACSA‐3, as recommended by the Mental Health Coordinating Council (2022). The Occupational Therapy Practice Framework: Domain and Process Third Edition (the Framework) and Fourth Edition (OTPF‐4) have updated some of the occupational categories including IADL and these changes were strongly considered while developing the DACSA‐3, as well as the major concepts of the Person–Environment–Occupation (PEO) Model (American Occupational Therapy Association [AOTA], 2014; AOTA, 2020; Law et al., 1996).

The Framework and OTPF‐4 are official documents of the AOTA that both present summaries ‘of interrelated constructs that describe occupational therapy practice’ (AOTA, 2014, p. S1; AOTA, 2020, p. 1). The Framework and OTPF‐4 both sort occupations into categories including IADL and list environmental and personal factors that affect occupational performance (AOTA, 2014; AOTA, 2020). The PEO Model states that ‘occupational performance is the outcome of the transaction of the person, environment, and occupation’, which is also a major concept that is represented in the Framework and the OTPF‐4 (Law et al., 1996, p. 16, AOTA, 2014, AOTA, 2020). The PEO Model helps the occupational therapist to widen their focus of assessment and intervention to incorporate the person, environment, and occupation when working in mental health practice (Strong & Rebeiro‐Gruhl, 2019). Other occupational therapy models acknowledge that IADL assessment and intervention are part of occupational therapy practice, so these models could also be used with the DACSA‐3 (Collister, 1994; Jaccoud‐Alexander, 1993).

The principles of mental health recovery were also considered while developing the DACSA‐3, particularly the inclusion of recovery goals and participant choice within the assessment (Andresen et al., 2011; Onken et al., 2007). Andresen et al. (2011, pp. 35–40) outlined ‘four component processes of recovery’ including ‘hope’, ‘responsibility’, ‘self and identity’, and ‘meaning and purpose’.

### 2.7 Qualitative review by professionals with lived mental health experience

Three content reviewers (professionals with lived mental health experience) were invited to participate in the qualitative review process, and two participated. Content reviewers were defined as peer workers who have a mental health condition or are carers or family members of a person with a mental health condition (Mental Health Coordinating Council, 2022; National Health and Medical Research Council & Consumers Health Forum of Australia, 2016). The two content reviewers were recruited using a recruitment letter, which was sent via email. Both content experts were known to the revision author of the DACSA‐3 via their place of employment. Both content reviewers worked in a public mental health service in Queensland, Australia, and were employed as a clinical assistant and consumer/carer consultant respectively.

The qualitative review process involved content reviewers to separately and individually complete a questionnaire and provide comments in the DACSA‐3 draft, which were sent to and from content reviewers via email including a copy of the Recovery Oriented Language Guide (Mental Health Coordinating Council, 2022). The content reviewers were asked to identify any areas in the DACSA‐3 where recovery oriented language was not included. Recovery oriented language is ‘respectful; non‐judgemental; clear and understandable; free of jargon, confusing data, and speculative comment; consistent with our body language; sincere in carrying a sense of commitment, hope and presenting the potential for opportunity; trauma‐informed; and strengths‐based’ (Mental Health Coordinating Council, 2022, p. 4). The initial draft of the DACSA‐3 was qualitatively reviewed in 2023 by the two content reviewers to ensure that recovery oriented language was included in the DACSA‐3 (Mental Health Coordinating Council, 2022; National Health and Medical Research Council & Consumers Health Forum of Australia, 2016). The data received from these content reviewers were collected and evaluated by the revision author of the DACSA‐3.

### 2.8 Content validity of the DACSA‐3

The content validity process of the DACSA‐3 used qualitative and quantitative methods as outlined by McKenzie et al. (1999). Ten content experts (occupational therapists with mental health experience) were invited to participate in the content review process, and nine participated. It is recommended that 5–10 content experts are required for content validity (Gilbert & Prion, 2016; Lawshe, 1975; Yusoff, 2019). Content experts were defined as occupational therapists who were working or had previously worked in the field of mental health. Content experts were recruited from the occupational therapy community in Australia using a recruitment letter, which was sent via email. Content experts were either known to the revision author of the DACSA‐3 via their place of employment or had been identified as suitable candidates during the literature search process. All content experts had experience working as occupational therapists and mental health clinicians with at least 2 years of experience. The revision author of the DACSA‐3 used reflexivity to acknowledge the potential for subjectivity, so was not involved as one of the content experts but was involved in the collection and evaluation of the data. Reflexivity is the process by which the researchers are aware that their own subjectivity can influence the research outcome (Olmos‐Vega et al., 2023).

The content experts were working in the following workplaces and had the following role titles at the time of this research project:

- Three in public mental health services in Queensland—two senior occupational therapists and one discipline lead of occupational therapy
- One in both a public mental health service and a private occupational therapy mental health service in Queensland—senior/private occupational therapist
- Two in private occupational therapy mental health services in Queensland—two senior private occupational therapists
- Two in public mental health services in Victoria—one occupational therapist and one senior occupational therapist
- One in a university occupational therapy department in New South Wales—associate professor of occupational therapy

**FIGURE 1:**
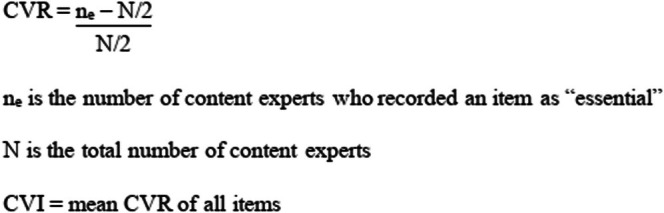
Equations for content validity ratio (CVR) and content validity index (CVI). *Note*: This figure outlines the CVR and CVI equations developed by Lawshe (1975).

The following steps were taken to establish content validity of the DACSA‐3:

1. The content of the second draft of the DACSA‐3 was qualitatively reviewed by a panel of nine content experts, and the recommendations were included in the third draft of the DACSA‐3. The qualitative review process involved the content experts separately and individually completing a questionnaire and providing comments in the draft of the DACSA‐3.
2. Following the qualitative review, the same panel of content experts was asked to separately and individually rate the appropriateness of each section and component of the DACSA‐3 quantitatively and qualitatively using a questionnaire including Lawshe's method scale:
  • 1 = Essential
  • 0 = Useful but not essential. Why?
  • 0 = Not necessary. Why? (Gilbert & Prion, 2016; Lawshe, 1975)
3. Step 2 was repeated using the fourth draft of the DACSA‐3, because content validity was not achieved for some items of the DACSA‐3. Lawshe's method recommended minimal values for content validity ratio (CVR) and content validity index (CVI) of 0.78 using nine content experts with a significance of P = 0.05 on a one‐tailed test (Lawshe, 1975).
4. The CVR and CVI were calculated using Lawshe's method (see Figure 1) to establish content validity of the DACSA‐3 (Gilbert & Prion, 2016; Lawshe, 1975).

All correspondence was sent to and from content experts via email.

## 3 RESULTS

### 3.1 Initial development of the DACSA‐3

The following structural changes were initially made to the DACSA‐3 when compared with the DACSA‐R:

- The DACSA‐3 uses the occupational category of IADL as per the Framework, though it could be separated into two occupational categories of IADL and Health Management as per the OTPF‐4 (AOTA, 2014; AOTA, 2020).
- The DACSA‐3 contains all the major concepts of the PEO Model including person, environment, occupation, and occupational performance (Law et al., 1996).
- The DACSA‐3 Initial Interview was retained and updated to obtain self‐reported information regarding the client's background, perception of task performance, motivation, environmental contexts, and recovery goals in relation to performing domestic and community tasks. If the client wishes, a support person can now be present and can contribute to the answers. The Initial Interview should not be used as a screening or stand‐alone assessment to determine or rate a client's level of performance, as Jaccoud‐Alexander (1993) found that the client or support person may underestimate or overestimate the client's level of performance. The information from the Initial Interview should not be used to rate any of the subtests in the DACSA‐3 Objective Assessment, but this information can be separately reported in the DACSA‐3 Occupational Therapy Report.
- The Supporting Interview was removed, as it was found by Jaccoud‐Alexander (1993) that a support person could not accurately rate the client's level of performance for the DACSA‐R; hence, it lacked construct validity.
- The 4‐point DACSA rating scale was retained (with definitions added) due to its previous correlation with Allen Cognitive Level Screen (ACLS) as found by Scanlan and Still (2013); however, its terminology was improved to use recovery oriented language (Mental Health Coordinating Council, 2022):
  ○ 4 = Fully Independent ➔ 4 = No Assistance: Able to fully perform the task without assistance from a support person.
  ○ 3 = Survival Independence ➔ 3 = Survival without Assistance: Able to perform the task without assistance from a support person, but there are limitations in performance or quality of the task.
  ○ 2 = Partially Dependent ➔ 2 = Partial Assistance: Requires partial assistance from a support person to complete the task.
  ○ 1 = Fully Dependent ➔ 1 = Full Assistance: Unable to perform the task and requires full assistance from a support person.
- The scoring criteria for each subtest in the DACSA‐3 Objective Assessment were aligned with the rating scale definitions to prevent discrepancies between DACSA‐3 ratings and occupational therapy clinical judgement regarding task performance (Grant, 2006).
- The Observation Checklist was renamed to the DACSA‐3 Objective Assessment Checklist and updated to include environmental and personal factors that may influence the client's task performance while performing the subtests in the DACSA‐3 Objective Assessment. The DACSA‐3 Objective Assessment Checklist was updated using the Framework, OTPF‐4, and PEO Model (AOTA, 2014; AOTA, 2020; Law et al., 1996). There was only one Objective Assessment Checklist that was developed for use with all DACSA‐3 subtests, rather than multiple Observation Checklists that were used for DACSA‐R subtests.
- The Information Form Completion subtest was renamed to Information Form.
- The DACSA‐3 Information Form was reformatted to resemble a form used in everyday life and to allow electronic completion by the client, which will be available as an additional resource to the DACSA‐3 manual (Grant, 2006).
- The Money Handling subtest was removed, as the ability to make payments is better assessed using observation during the Grocery Shopping subtest (Grant, 2006).
- The DACSA‐3 Budgeting and Meal Planning worksheets were reformatted to make them easier to use and to allow electronic completion for the client, which will be available as additional resources to the DACSA‐3 manual (Grant, 2006).
- A sample bill was included in the Paying Bills subtest and includes electronic bill paying options, which will be available as an additional resource to the DACSA‐3 manual (Grant, 2006).
- The Making and Keeping Appointments subtest was renamed to Appointment Attendance.
- The Public Transport subtest was renamed to Transport to include other means of transport.
- The Telephone Use subtest was renamed to Electronic Communication to include a wider range of communication technologies that are currently used in everyday life.
- The Postal Services subtest was removed due to decreased personal reliance on postal services in everyday life for outgoing mail (Grant, 2006).
- An Other Tasks subtest was added for the occupational therapist to consider additional domestic and community tasks that are not included as one of the 14 DACSA‐3 subtests, which can be assessed using observation and the DACSA‐3 rating scale.
- All of the DACSA‐3 Objective Assessment subtests were updated to consider changes in and use of technology (Grant, 2006). The technological IADL advancements that were incorporated into the DACSA‐3 included: computer, tablet, smart phone, or other device use including internet and apps; electronic form completion; online banking and payment options; online bill payment; meal planning apps and online meal delivery; smart phone or other device payment options; online grocery shopping; robotic cleaning devices; ride‐sharing services; electronic scooter use; videoconferencing and video calls; SMS, email, and online messaging; Internet search engine use; and voice recognition and artificial intelligence use.
- The DACSA‐3 Objective Assessment Recording Sheet was included to assist the occupational therapist with recording working notes and observations.
- The DACSA‐3 Occupational Therapy Report was improved to include the following headings: Reason for Referral, Recovery Goals, Background Information, Client's Perceived Strengths, Client's Perceived Weaknesses, Environmental Factors Affecting Performance, Personal Factors Affecting Performance, Results from DACSA‐3 Objective Assessment, and Recommendations and Intervention Plan (Grant, 2006).
- All DACSA‐3 assessment forms and report were reformatted to allow electronic completion by the occupational therapist, which will be available as additional resources to the DACSA‐3 manual (Grant, 2006).

### 3.2 Results from the review by professionals with lived mental health experience

Overall, the professionals with lived mental health experience agreed with the recovery oriented language that was used in the initial draft of the DACSA‐3. One suggestion was to use, ‘clients living with schizophrenia’ rather than ‘clients with schizophrenia’ in History and Development of the DACSA‐3. The Recovery Oriented Language Guide was reviewed and the following language was recommended:

- ‘person with a mental health condition’
- ‘a person who has been diagnosed with …’
- ‘a person diagnosed with …’

(Mental Health Coordinating Council, 2022).

Amendments were made to ensure the above language recommendations were included in the second draft of the DACSA‐3.

### 3.3 Results from the qualitative review by content experts

The content experts qualitatively reviewed the second draft of the DACSA‐3 and made the following recommendations for changes:

- To retain 'fully independent' and 'survival independence' in the DACSA‐3 rating scale. The meaning of independent or independence does not have a singular definition in occupational therapy and it could mean that the person can:
  1. perform the task without assistance,
  2. perform the task with assistive equipment or modified techniques,
  3. perform the task by directing someone else to perform the task for them (AOTA, 2014; AOTA, 2020; Collins, 2017; Hinojosa, 2002).
  A decision was therefore made to retain the following changes to the DACSA‐3 rating scale due to clearer definitions:
  ○ 4 = No Assistance: Able to fully perform the task without assistance from a support person.
  ○ 3 = Survival without Assistance: Able to perform the task without assistance from a support person, but there are limitations in performance or quality of the task.
- A suggestion was made to change the word ‘survival’ with ‘adequate performance’ in the DACSA‐3 rating scale. The word ‘survival’ is unique to the DACSA rating scale and was retained in the DACSA‐3 because of the previous correlation with ACLS (Scanlan & Still, 2013). Three theoretical concepts also helped to guide this decision:
  ○ Participation in personal ADL/self‐care and IADL is required for survival in the community (Christiansen & Hammecker, 2001).
  ○ Humans are able to adapt to the environment and need to engage in purposeful occupations to survive in the community (Wilcock, 1993).
  ○ In mental health recovery, it is important for the person to not ‘only survive but to thrive’ in the community when faced with challenges and difficulties (Onken et al., 2007, p. 15).
- The DACSA‐3 Objective Assessment Checklist was updated using the Factors influencing Occupational Performance checklist (O'Sullivan & Fitzgibbon, 2024).
- The Information Form subtest was renamed to Form Completion.
- The budgeting component was removed from the Meal Planning subtest because an ‘appropriate budget’ for a meal may vary between households.
- The making of a shopping list was moved from the Meal Planning subtest to the Grocery Shopping subtest because it was better related to this subtest.
- The DACSA‐3 Spaghetti Bolognese Recipe was included as an option for the Meal Preparation subtest.
- Cultural factors were included for the Meal Planning and Meal Preparation subtests for balanced, healthy meals.
- The Personal Presentation subtest was removed because it was measuring the construct of ADL or self‐care, so it did not fit in the occupational category of IADL and the subtest contained subjectivity (AOTA, 2014, 2020).
- The Appointment Attendance subtest was renamed to Attending Appointments.
- Headings in the DACSA‐3 Occupational Therapy Report were renamed:
  ○ Client's Perceived Strengths to Client's Self‐Reported Strengths
  ○ Client's Perceived Weaknesses to Client's Self‐Reported Difficulties

### 3.4 Content validity of the DACSA‐3

The first attempt to establish content validity of the DACSA‐3 did not score a CVR of greater than or equal to 0.78 for all items (see Table 1). Lawshe (1975) identified that eight out of nine content experts must rate an item as essential to achieve a minimum CVR of 0.78. The following three items scored a CVR of 0.11, 0.11, and 0.33, respectively, and were deemed not essential and removed from the DACSA‐3:

- History and Development of the DACSA‐3
- Other Tasks subtest
- DACSA‐3 Occupational Therapy Report

**TABLE 1:** First attempt to establish content validity using CVR.

Item name	Content experts (n = 9)									CVR
	1	2	3	4	5	6	7	8	9	
History and Development of the DACSA‐3	0	0	0	1	1	1	1	1	0	0.11
DACSA‐3 Administration Instructions	1	1	1	1	1	1	1	1	1	1.00
DACSA‐3 Initial Interview	1	1	1	1	1	1	1	1	1	1.00
DACSA‐3 Objective Assessment Checklist	1	0	1	1	1	1	0	1	1	0.56
Form Completion	1	1	1	1	1	1	1	0	1	0.78
Banking	1	1	1	1	1	1	1	1	1	1.00
Budgeting	1	1	1	1	1	1	1	1	1	1.00
Paying Bills	1	1	1	1	1	1	1	1	1	1.00
Meal Planning	1	1	1	1	1	1	1	1	1	1.00
Grocery Shopping	1	1	1	1	1	1	1	1	1	1.00
Meal Preparation	1	1	1	1	1	1	1	1	1	1.00
House Cleaning	1	1	1	1	1	1	1	0	1	0.78
Laundry	1	1	1	1	1	1	1	0	1	0.78
Medication Management	1	1	1	0	1	1	1	1	1	0.78
Simple First Aid	1	1	1	1	1	1	1	1	1	1.00
Attending Appointments	1	1	1	1	1	1	1	0	1	0.78
Transport	1	1	1	1	1	1	1	1	1	1.00
Electronic Communication	1	1	1	1	1	1	1	1	1	1.00
Other Tasks	1	0	1	0	0	1	1	0	1	0.11
DACSA‐3 Objective Assessment Recording Sheet	1	1	1	0	1	1	0	0	1	0.33
DACSA‐3 Occupational Therapy Report	1	0	1	0	1	1	1	0	1	0.33

*Note*: Lawshe's method scale: 1 = *essential*, 0 = *useful but not essential*, 0 = *not necessary*, and CVR threshold ≥0.78 (Lawshe, 1975).

An introduction was added to the DACSA‐3 Administration Instructions and was renamed to DACSA‐3 Introduction and Administration Instructions. The DACSA‐3 Objective Assessment Checklist and DACSA‐3 Objective Assessment Recording Sheet only recorded a CVR of 0.56 and 0.33, respectively, but were not removed from the DACSA‐3, because they were deemed essential by the revision author. The DACSA‐3 Objective Assessment Checklist was developed using occupational therapy models and theories, and the DACSA‐3 would lose meaning without it. Some of the content experts stated they could record the results of the DACSA‐3 using their own methods or format, but the revision author believed it would be particularly helpful for occupational therapists to have the option of using the DACSA‐3 Objective Assessment Recording Sheet. The DACSA‐3 Objective Assessment Checklist was renamed to DACSA‐3 Context List (to align with terminology in the Framework and OTPF‐4) and reviewed and amended using recommendations from content experts and literature sources (AOTA, 2014, 2020; Law et al., 1996; O'Sullivan & Fitzgibbon, 2024). The DACSA‐3 Objective Assessment Recording Sheet was renamed to DACSA‐3 Objective Assessment Record and amended to make it a more concise method for recording the results from the DACSA‐3 Objective Assessment.

The final attempt to establish content validity of the DACSA‐3 produced a CVR of greater than or equal to 0.78 for all items (see Table 2), which exceeded the minimum recommended threshold CVR value of 0.78 for content validity to be achieved for assessment items (Lawshe, 1975). The CVI for the DACSA‐3 was calculated as 0.98 (see Table 2), which exceeded the minimum recommended threshold CVI value of 0.78 for content validity to be achieved for the whole assessment (Lawshe, 1975). Content validity of the DACSA‐3 was established in 2024.

**TABLE 2:** Final attempt to establish content validity using CVR and CVI.

Item name	Content experts (n = 9)									CVR
	1	2	3	4	5	6	7	8	9	
DACSA‐3 Introduction and Administration Instructions	1	1	1	1	1	1	1	1	1	1.00
DACSA‐3 Initial Interview	1	1	1	1	1	1	1	1	1	1.00
DACSA‐3 Context List	1	1	1	1	1	1	1	1	1	1.00
Form Completion	1	1	1	1	1	1	1	1	1	1.00
Banking	1	1	1	1	1	1	1	1	1	1.00
Budgeting	1	1	1	1	1	1	1	1	1	1.00
Paying Bills	1	1	1	1	1	1	1	1	1	1.00
Meal Planning	1	1	1	1	1	1	1	1	1	1.00
Grocery Shopping	1	1	1	1	1	1	1	1	1	1.00
Meal Preparation	1	1	1	1	1	1	1	1	1	1.00
House Cleaning	1	1	1	1	1	1	1	1	1	1.00
Laundry	1	1	1	1	1	1	1	1	1	1.00
Medication Management	1	1	1	1	1	1	1	1	1	1.00
Simple First Aid	1	1	1	1	1	1	1	1	1	1.00
Attending Appointments	1	1	1	1	1	1	1	0	1	0.78
Transport	1	1	1	1	1	1	1	1	1	1.00
Electronic Communication	1	1	1	1	1	1	1	1	1	1.00
DACSA‐3 Objective Assessment Record	1	0	1	1	1	1	1	1	1	0.78
CVI										0.98

*Note*: Lawshe's method scale: 1 = *essential*, 0 = *useful but not essential*, 0 = *not necessary*, and CVR/CVI threshold ≥ 0.78 (Lawshe, 1975).

All of the assessment items that were deemed as not essential by content experts during the first and final attempts to establish content validity were recorded as ‘useful but not essential’.

## 4 DISCUSSION

### 4.1 Revision and content validity of the DACSA‐3

The DACSA‐3 is a revised, contemporary occupational therapy assessment of IADL, which has content validity (Woodroffe et al., 2025). A combination of guidelines for test development and content validity was used to revise the DACSA‐3 (Benson & Clark, 1982; Cronje et al., 2022; Lawshe, 1975; McKenzie et al., 1999). McKenzie et al. (1999) outlined a detailed step‐by‐step process to establish content validity using qualitative and quantitative processes, including Lawshe's method using CVR and CVI. Lawshe's method using content experts has been widely accepted across many disciplines as a robust method for establishing content validity (Ayre & Scally, 2014; Wilson et al., 2012). Gilbert and Prion (2016) added ‘Why?’ to Lawshe's method scale, which was not included in the original version of the scale. The introduction of ‘Why?’ was helpful during the content validity process of the DACSA‐3, as content experts were able to provide reasons (qualitative data) why they scored (quantitative data) an item as non‐essential. As a result, this process only needed to be completed twice and made the content validation process more efficient and timely.

The Test Adaptation Report Standards (TARES) has been used to compare and summarise of the content revision and validity evidence between the DACSA‐R and the DACSA‐3, which can be found in Table 3 (Collister & Alexander, 1991; Iliescu et al., 2023; Woodroffe et al., 2025).

**TABLE 3:** Comparison and summary of the content revision and validity evidence between the DACSA‐R and the DACSA‐3.

Checklist item	DACSA‐R	DACSA‐3
Intended population	Adult with a psychiatric illness (aged 16+)	Person with a mental health condition (aged 16+)
Purpose and intended use	Occupational therapy assessment of essential community living skills	Occupational therapy instrumental activities of daily living (IADL) assessment
Need for adaptation	Language was not recovery oriented Lacking evidence for validity of Supporting Interview Subtest items out of date or no longer relevant	Includes recovery oriented language Supporting Interview was removed Subtest items were updated or removed if no longer relevant
Coverage of adaptation	Initial Interview, Supporting Interview, and 17 Objective Subtests including Observation Checklists	Initial Interview and 14 Objective Subtests including Context List
Instructions and scoring rubrics	Standardised instructions included 4‐point rating scale	Standardised instructions included 4‐point rating scale was improved by including definitions and recovery oriented language
Test manual	Manual was only available in print format	Manual is freely available online in PDF format with additional electronic resources
Reliability	Evidence was found for internal consistency, inter‐rater reliability, and test–retest reliability	Reliability has not yet been tested
Validity evidence	Evidence was found for content validity, factor analysis, construct validity, concurrent validity, discriminant validity, and predictive validity	Only content validity has been established. Other forms of validity have not yet been tested
Norms	No normative data have been collected	No normative data have been collected

*Note*: Comparison and summary using Test Adaptation Report Standards (TARES) (Collister & Alexander, 1991; Iliescu et al., 2023; Woodroffe et al., 2025).

### 4.2 Time to administer and screening for the DACSA‐3

Hitch et al. (2007) and Grant (2006) both reported that time to administer the DACSA‐R impacted its usability in occupational therapy practice. The time to administer the DACSA‐3 has been shortened due the removal of the Supporting Interview and the reduction of 17 to 14 subtests, but as a result, it no longer contains a screening tool. It has also been emphasised that only part of the DACSA‐3 has to be completed, considering the benefits to the client and their recovery goals (Woodroffe et al., 2025). Exact administration time has not been specified in the DACSA‐3 administration instructions, as it will be dependent on whether whole or part of the assessment is completed (Woodroffe et al., 2025).

Collister and Alexander (1991) hypothesised that if data from the Initial Interview correlated with the data from the Supporting Interview, then the corresponding objective subtest did not necessarily need to be completed, hence operating as a screening tool for the DACSA‐R. Jaccoud‐Alexander (1993) found that both the client and support person could not accurately rate the client's level of performance; therefore, the Initial Interview and Supporting Interview from the DACSA‐R should not be used as a screening tool. Grant (2006) found that less than 50% of occupational therapists (n = 36) reported using both the Initial Interview and Supporting Interview from the DACSA‐R, so it was likely not being used a screening tool by these occupational therapists. Scanlan and Still (2013) found a correlation between ACLS scores and a modified DACSA‐R rating scale, leading to a hypothesis for further research that the ACLS could be used as a screening tool for the DACSA‐3.

### 4.3 Importance of the DACSA‐3

The DACSA‐3 assesses performance in IADL, which has been found to be an important health indicator (Portela et al., 2020). As per previous versions of the DACSA, assessment results from the DACSA‐3 may be used to assist clinical decision making, such as discharge planning, rehabilitation needs, support needs, and determining safe accommodation for clients with a mental health condition (Collister & Alexander, 1991; Woodroffe et al., 2025). The DACSA was previously identified as an occupational therapy assessment for use with the National Disability Insurance Scheme (NDIS) in Australia; therefore, the DACSA‐3 could be used with people with a mental health condition who receive NDIS funding (Winkler et al., 2022).

### 4.4 Current limitations of the DACSA‐3 and this study

The community involvement used to develop the DACSA‐3 was limited to two professionals with lived mental health experience, though the Australian guidelines for community involvement in research do not specify a minimum requirement for community involvement (National Health and Medical Research Council & Consumers Health Forum of Australia, 2016). Cultural factors were deemed important in the DACSA‐3, but there was no consultation with Aboriginal and Torres Strait Islander people or other multicultural groups in Australia during the revision process. Future revisions of the DACSA should consider the involvement of reviewers from these cultural groups.

The process to establish content validity of the DACSA‐3 may have been affected by bias. The responses from the content experts were subjective, so the DACSA‐3 content validity process was potentially subjected to bias from the content experts' own beliefs and ideas (Rubio et al., 2003; Sireci & Faulkner‐Bond, 2014; Zamanzadeh et al., 2015). The process to establish content validity for the DACSA‐3 was lengthy, so the content experts may have agreed that some items were essential due to fatigue or reduced motivation in the process (Delgado‐Rico et al., 2012). Some of the content experts for the DACSA‐3 were known to the revision author, so their responses may have been affected by social desirability bias to please the revision author (Sireci & Faulkner‐Bond, 2014). Sireci (1998) implied that judgemental methods of content validity (e.g. Lawshe's method) have a problem with bias, which could be avoided via statistical methods (e.g. analysis of item scores from an administered assessment).

Content validity was required, but not enough to establish validity of the DACSA‐3; therefore, further testing to establish construct validity will be required to determine whether the DACSA‐3 actually measures occupational performance of IADL (Cronbach & Meehl, 1955; Messick, 1998; Santoso et al., 2022; Sireci, 1998). Internal consistency should also be measured to determine how well the DACSA‐3 measures occupational performance of IADL (Fenn et al., 2020). These studies were deemed beyond the scope of this study, so construct validity and internal consistency of the DACSA‐3 have not yet been tested.

It is suggested that a pilot and full study be conducted with a sample of participants with a mental health condition (e.g. affective, psychotic, or anxiety disorder) including Aboriginal and Torres Strait Islander people or other multicultural groups to establish construct validity and internal consistency of the DACSA‐3. Other future studies may also involve inter‐rater reliability, test–retest reliability, and predictive validity for accommodation suitability, as these were previously conducted for the DACSA‐R (Collister, 1994; Jaccoud‐Alexander, 1993). Total ratings for the DACSA‐3 should not be currently calculated, as no normative data have been collected (Woodroffe et al., 2025). Normative data could however be collected to allow for the DACSA‐3 to be developed as a summed scale, which was suggested for the DACSA‐R as it yielded a high alpha coefficient of 0.89 (Jaccoud‐Alexander, 1993).

The DACSA‐3 may not be accepted as a fully standardised assessment until it has been tested for construct validity and reliability, and normative data are collected. A standardised assessment has consistent administration and scoring, has gone through a process of testing for validity and reliability, and has well‐defined norms or criteria (American Psychological Association, 2023; Marazita et al., 2024). The DACSA‐3 can initially be used as a non‐standardised assessment until it becomes a fully standardised assessment through further testing (Luebben & Royeen, 2005, as cited in Classen & Velozo, 2024). The DACSA‐R was inherently being used as a non‐standardised assessment because the research regarding its validity and reliability was never formally published or made available to the occupational therapy community (Collister, 1994; Collister & Alexander, 1991; Jaccoud‐Alexander, 1993). Occupational therapists generally use a combination of standardised and non‐standardised assessments in clinical practice (Classen & Velozo, 2024; Marazita et al., 2024).

One of the major challenges of establishing construct validity of the DACSA‐3 is that the environment, society, and IADL change over time, which may identify a need to revise the assessment again (Classen & Velozo, 2024; Fessler et al., 2022). It appears that the authors of the Kohlman Evaluation of Living Skills (KELS), Fourth Edition, may have also experienced similar difficulties in re‐establishing construct validity of the assessment due to changes in living skills over time (Kohlman Thomson, 2020). For this reason, there may be rationale to leave the DACSA‐3 as a non‐standardised assessment to allow for easier revisions and real‐life application. Coelho et al. (2005) stated that non‐standardised assessments may be better at assessing real‐life situations and activities.

## 5 CONCLUSION

The DACSA‐3 is a revised, contemporary occupational therapy assessment of IADL, which has content validity. The CVR and CVI exceeded the minimum recommended CVR and CVI thresholds, and thus, the content validity of the DACSA‐3 was established. The process to revise the DACSA‐3 involved collecting and reviewing past research; updating content based on research, occupational therapy theories and technology changes; and consulting professionals with lived mental health experience and content experts. Modifications were made to the DACSA‐3 to reduce the administration time, but it no longer contains a screening tool. It may be beneficial to conduct further research for construct validity and reliability and to determine if the ACLS could be used as a screening tool for the DACSA‐3.

The DACSA‐3 manual including administration instructions and resources can be downloaded for free from https://neilwoodroffe.github.io/DACSA-3/.

## AUTHOR CONTRIBUTIONS

The first author completed the project design, project development, data collection, analysis process, and prepared the draft of the manuscript. All the authors reviewed the project design, project development, data collection, analysis process, and edited the final copy of the manuscript.

## CONFLICT OF INTEREST STATEMENT

The authors report no conflict of interest.

## DECLARATION OF USE OF ARTIFICIAL INTELLIGENCE

The plain language summary was entered into ChatGPT with the question, ‘Is the following paragraph Flesch–Kincaid Level 8’. The recommendations were added, checked, refined, and confirmed by the authors before inclusion.

## Data Availability

The data that support the findings of this study are available from the corresponding author upon reasonable request.
